# The Christmas Season

**Published:** 1916-12

**Authors:** 


### The Christmas Season.

We are inclined to smile at the little fellow in Eugene Field's poem who was just as good as he could be just before Christmas, although admitting that at other seasons of the year there were no flies on him. But after all that is a pretty fair description to apply to the old as well as the young; and it is well enough. If we can't be as good as can be the rest of the year, it is at least commendable to make the effort just before Christmas.

Christmas is a season of good will and good cheer. We are all willing to admit that the rest of the year is just as fitted for these good attributes of character as Christmas, but somehow, although we want to dispense cheerfulness and helpfulness, in our hurry or in our selfishness we often forget to do it. Since people are not perfect, it is a good thing that there is a time of the year when the heart gets a little warmer than at other seasons and when there comes a longing to live closer to one's fellowmen and to recognize the universal kinship of all God's people. By common consent, Christmas is that time; the time when people are less selfish and more altruistic; when they are less avaricious and more generous; when they are less vindictive and more forgiving.

At Christmas time the people are accustomed to review the past and take an inventory of themselves to see what progress they have made, what mistakes have retarded their development and what hindrances have been in the way of success. Most people when at their best want to do right, but temptations constantly beset them. The greatest obstacle in the way of the masses is ignorance and lack of the mental energy necessary to overcome it. Professional people especially are not studious enough, and physicians are not exceptions to the rule. The physician who has failed to make progress the past year can in most instances attribute it to a lack of constant and persistent study. What preacher could hold his congregation without devoting much time to study; what teacher could advance his pupils if satisfied to stop his own development at graduation; what lawyer could succeed without giving time to the text-books over each case? Too many physicians think that if they are only busy in their practice that they need not spend time studying text-books and journals. These soon find themselves falling behind in their profession, and wonder why it is.

Practice helps to keep the wits sharpened, but it cannot entirely take the place of reading and study. Look back over the past year and try to calculate about what proportion of your time you have given to reading and study. Have you had any system about it, or have you just read between times? Have you read with a view to your own improvement, or have you just read for pastime? Have you really worked at study with any enthusiasm, or have you read with an I-know-it-better sort of a feeling?

The opinion of the TEXAS MEDICAL JOURNAL is that every medical man should set apart regular hours for reading, just as he has regular office hours for seeing his patients. He should honestly try to keep those hours and only let urgent business keep him from it. Of course, no physician in practice can say that any hour belongs absolutely to him, but where he finds his study time taken in practice, he can substitute another study period, and should do it. The professional man who determines that he will study just as earnestly while in the practice of his profession as he did while working for a diploma will never have occasion to complain of a lack of patronage. The man who allows his professional mind to shrink deserves to lose patronage. In making your inventory of the past year's successes and failures, keep that in mind.

Another thing should be considered. Have you tried to keep in close touch with your fellowmen during the past year? Have you rubbed shoulders with them and always found a sympathetic touch? Or have you enclosed yourself in your professional dignity and held aloof from people except as the spirit of association has moved you? No man can succeed as he should unless he is able to get something from association with others. If you have failed to meet with your brother physicians in the exchange of opinions, if you have even failed to mingle socially with your fellowmen, you have deprived yourself of one of the most helpful methods of self-improvement. Resolve to know more about those around you, to try to be one of them.

---

Do you know that the United States Public Health Service is the nation's first line of defense against disease?

